# Association between Physical Activity and Telomere Length in Women with Breast Cancer: A Systematic Review

**DOI:** 10.3390/jcm11092527

**Published:** 2022-04-30

**Authors:** Jihee Min, Ji Young Kim, Ji Yeong Choi, In Deok Kong

**Affiliations:** 1 Department of Convergence Medicine, Wonju College of Medicine, Yonsei University, Wonju 26426, Korea; jihee8700@yonsei.ac.kr (J.M.); 0527cjy@yonsei.ac.kr (J.Y.C.); 2 Yonsei Institute of Sports Science and Exercise Medicine (YISSEM), Wonju 26426, Korea; 3 Department of Physiology, College of Medicine, Korea University, Seoul 02841, Korea; skyditto01@korea.ac.kr

**Keywords:** breast cancer, physical activity, exercise, telomere length/single-copy gene (T/S) ratio

## Abstract

The association between physical activity and telomere length (TL) has been continuously reported. However, the interplay of physical activity and TL among women with breast cancer has not been elucidated. Thus, the purpose of this systematic review was to synthesize the evidence for the association of physical activity with TL in women with breast cancer. Systematic searches were conducted to identify quantified studies using MEDLINE, EMBASE, Cochrane Central Register of Controlled Trials, Web of Science, and Clinical Trials.gov. Five studies were included in this systematic review. Three of the five studies reported that physical activity has a significant relationship in delaying TL shortening, but others observed no association between physical activity and TL in breast cancer survivors. Although the heterogeneous studies acted as limitations in drawing clear conclusions, physical activity strategies show encouraging impacts in delaying TL shortening. To understand the effects of physical activity on TL shortening in breast cancer survivors, further studies are needed considering the tissue site, treatments for breast cancer, DNA extraction methods, and tools for measuring physical activity.

## 1. Introduction

Telomeres are nucleoprotein structures located at the end of human eukaryotic chromosomes, and they protect against genome instability and damage [1]. Telomeres shorten with each cell cycle in most cells; thus, telomere length (TL) represents the proliferative history of the cell [2,3]. Once telomeres approach a critical size, cell-signaling events occur and induce cellular senescence or apoptosis [4].

TL is negatively associated with biological aging and age-related diseases such as diabetes [5], dementia [6], cancer [7,8], and chronic psychiatric disorders [9,10]. In particular, there is growing evidence of the association between breast cancer and telomere length. Previous studies reported that an increase in inflammatory markers [11], an increased mitochondrial dysfunction [12], and the accumulation [13] of reactive oxygen species not only increase the risk of breast cancer but have a close relationship with telomere shortening. Furthermore, it has been shown that telomere shortening is related to the mutation of the breast cancer susceptibility gene 2 (BRCA2), which is highly associated with the recurrence of breast cancer [14]. A recent review reported that TL could be a valuable prognostic marker of breast cancer despite major methodological differences in measuring TL [15].

Physical activity and exercise are important modifiable factors in breast cancer prevention [16], prognosis [17,18], and mortality [19]. They also have a strong association with delayed telomere shortening. A meta-analysis revealed that active individuals had significantly longer telomeres compared to inactive individuals regardless of the intensity of exercise (mean difference 0.15, 95% CI 0.05–0.24, I^2^ = 99%) [20]. However, studies on the effect of physical activity or exercise on TL in breast cancer survivors are limited, and inconsistent results have been obtained [21,22,23,24,25].

Therefore, we aimed to comprehensively analyze the association and effect of physical activity or exercise on delaying telomere shortening in women with breast cancer.

## 2. Materials and Methods

### 2.1. Search Strategy

The current study protocol was registered in the PROSPERO database (No. CRD42021253013), and the study was conducted according to the Preferred Reporting Items for Systematic Review and Meta-analyses (PRISMA) checklist [26]. We searched the following databases: MEDLINE, EMBASE, Cochrane Central Register of Controlled Trials (CENTRAL, The Cochrane Library), Web of Science, and http://ClinicalTrials.gov (accessed on 23 April 2021) from inception up to April 2021. We also reviewed the reference lists of recent systematic reviews.

All articles that assessed the relationship between physical activity and TL or the effects of exercise on TL in breast cancer survivors were included in this review. The search was performed using the following terms: “breast cancer” AND “physical activity” OR “exercise” AND “telomere length”. The full search strategy is available in the Table S1. 

This section may be divided by subheadings. It should provide a concise and precise description of the experimental results, their interpretation, as well as the experimental conclusions that can be drawn.

### 2.2. Eligibility Criteria

We included studies that (1) were quantitative studies, including cross-sectional, case-control, and intervention studies, and (2) reported TL as mean ± standard deviation or median (interquartile range) or TL/single-copy gene (T/S) ratio.

### 2.3. Data Extraction and Quality Assessment

Two reviewers (J.M. and J.K.) independently screened all titles, abstracts, and full texts to identify studies based on the inclusion criteria using the Covidence systematic review software (Veritas Health Innovation, Melbourne, Australia, www.covidence.org accessed from 25 April 2021 to 7 May 2021). Any disagreement between the reviewers was resolved through discussion. The information obtained from each selected study included the first author’s name, year of publication, study design, population, patients’ ages, method of TL evaluation, tissue where TL measurement was conducted, tool for assessing physical activity, intervention (i.e., type of exercise, frequency, intensity, time), main results, and any other information considered relevant.

The quality of eligible studies was assessed with the Newcastle-Ottawa Scale (NOS). Considering the different study designs, we used three versions of the NOS for randomized controlled trials, cross-sectional studies, and case-control studies, respectively [27]. NOS scales for non-randomized studies and randomized controlled trials consist of three domains: selection, comparability, and outcome/exposure. We defined NOS ≥ 7 as high quality and 4 < NOS < 7 as low to moderate quality.

## 3. Results

### 3.1. Study Search and Selection

**Figure 1:**
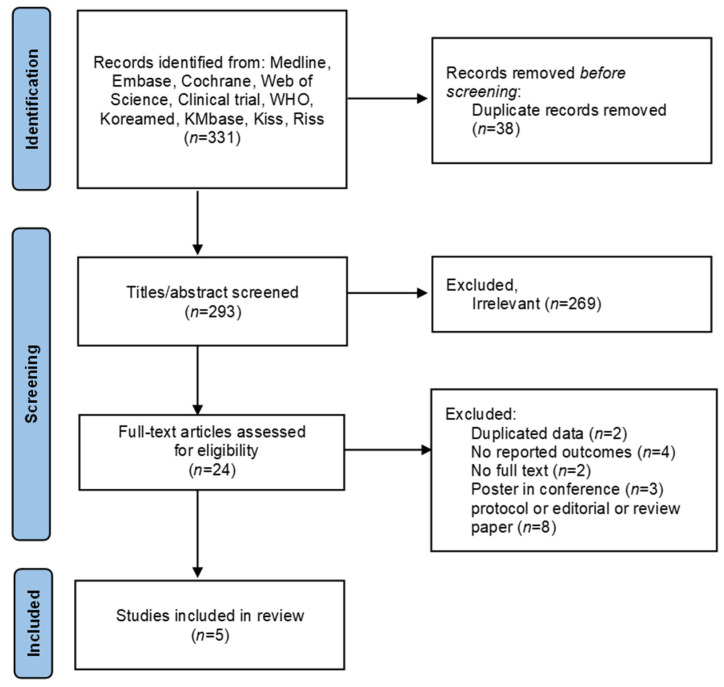
PRISMA flow diagram for systematic review.

A total of 331 studies were retrieved using the search strategy (Table S1), of which 293 were retained after the title and abstract were screened and duplicates were removed. Of these 293 studies, 269 were excluded because they were considered non-relevant. After a full-text review of the 24 papers considered relevant, 19 studies were excluded (2 with duplicate data, 4 without TL results, 3 conference abstracts, and 8 protocol, editorial papers, and review papers). Finally, 5 studies (2099 participants) were included in this review (Figure 1).

### 3.2. Description of the Studies and Quality Assessment

Table 1 shows the summary of the included studies and the results of the quality assessment. Of the five studies, two were randomized controlled trials, two were cross-sectional studies, and one was a case-control study. For the TL assessment, three studies used quantitative-polymerase chain reaction, one used a fluorescence in situ hybridization assay, and one used the terminal restriction fragment method. The mean NOS score was 6.2 (range: 5–7), and all included studies had moderate to high scores (Table 1).

### 3.3. Effects of Exercise on Telomere Length in Women with Breast Cancer

Of the two randomized controlled trial studies, one reported that exercise intervention had a significant effect on delaying TL shortening, while the other reported no intervention effect on TL (Table 2).

Santa-Maria et al. [21] conducted a 12-month web-based exercise intervention on 96 obese breast cancer survivors treated in their region. The intervention group received a total of 21 phone consultations consisting of 20 min weight-loss counseling once a week for the first 3 months and once a month for the remaining 9 months. Professional exercise coaches monitored the exercise log, meal log, and weight on the web-based platform. The control group was advised by medical staff to maintain a healthy weight and was provided with weight loss consultations from exercise professionals once. After a 6-month intensive lifestyle intervention, TL in the intervention group reduced by 3% while TL in the usual care group reduced by 5% (*p* > 0.05). In addition, when classifying by stage of breast cancer, stage 0 or 1 showed a significantly lower reduction in TL for the intervention group than for the control group after 6 months (*p* < 0.05). However, there were no significant differences between the groups for breast cancer survivors who were diagnosed with cancer exceeding stage 2.

Sanft et al. [24] conducted an exercise intervention on 151 obese breast cancer survivors who had been involved in dietary regulation and exercise to achieve weight loss over 6 months. The weight loss strategy included attaining 150 min/week of moderate-intensity activity and 10,000 steps per day as well as reducing calories to 1200–2000 kcal/day. In addition, the patients were advised to reduce dietary fat to <25% of the total energy intake and participate in behavior modification sessions once or twice every month. No significant difference was noted in the change in TL between the intervention and control groups. In addition, the TL was not significantly associated with weight loss or chemotherapy.

### 3.4. Association between Physical Activity and Telomere Length in Women with Breast Cancer

Three cross-sectional or case-control studies were conducted to analyze the associations between physical activity or exercise and TL among breast cancer patients or survivors (Table 3). Two studies showed a significant quantitative correlation between physical activity and TL in breast cancer patients, while one reported that no association existed between exercise and TL. A study of 162 breast cancer patients, who were scheduled to undergo surgery, identified linear trends in the positive association between total physical activity and TL (*p* < 0.05). Moreover, linear trends for increasing TL were observed for physical activity at the transportation-related physical activity (*p* < 0.05). However, age, stage of cancer, and menopausal status were not significantly associated with TL [27]. Garland et al. [22] used the International Physical Activity Questionnaire (IPAQ) to analyze the association between physical activity and TL among 392 postmenopausal breast cancer survivors. Compared to those who participated in any physical activity, breast cancer survivors who participated in moderate to vigorous physical activity had significantly longer TL (*p* < 0.05). However, a case-control study by Qu et al. [25] announced no significant association between TL and exercise.

## 4. Discussion

This systematic review was conducted to clarify the association between physical activity and TL in breast cancer patients and survivors. Five papers were reviewed and there was difficulty in synthesizing the research results because of heterogeneity between the studies.

Previous studies reported that physical activity may compensate for shortened TL in cancer survivors [24,28,29,30]. Physical activity may alleviate the decrease in TRF-2 [31]. Furthermore, exercise can help reduce oxidative stress [32] and inflammatory responses [21] which contribute to telomere damage. The stress response can damage cells, releasing damaged cell components, while exercise promotes autophagy [33]. Increased physical activity has been reported to reduce factors related to oxidative stress and inflammation, such as high-sensitivity C-reactive protein, insulin resistance, interleukin-6, tumor necrosis factor-alpha, granulocyte colony-stimulating factor, and F2-isoprostane [11,34,35].

Although we examined the correlation between physical activity and TL of breast cancer patients and survivors, it was challenging to derive consistent results owing to interstudy heterogeneity.

There are three prominent reasons for why the results differed among the studies.

First, the effect of the mediator variables on the length of the telomeres should be considered. Factors such as ethnicity [36], cancer stage distribution [37], radiation therapy [38], chemotherapy [39], and menopause [40] affect breast cancer prognosis and treatment and are also related to TL. In addition, breast cancer survivors experience a mix of both cancer- and metabolic- related problems. Heo, J et al. [41] reported that 36.7% of breast cancer survivors had been newly diagnosed with comorbidities such as diabetes, hypertension, and metabolic syndrome.

Second, we could consider the difference in intervention effectiveness across studies. In our extracted intervention studies, the participants of both studies [21,24] experienced significant weight loss. While 20% of the participants showed a reduction in their body fat by more than 5% in the study by Sanft et al. [24], change in body composition was not reported in the study by Santa-Maria et al. [21]. If the participants’ weight loss in the Santa-Maria et al. [21] was derived from muscle reduction, the effect could be considered relatively weak. As breast cancer prognosis is highly related to body fat and muscle, it is necessary to carefully monitor body composition rather than simply focus on weight loss.

Third, there were differences in the evaluation of TL and physical activity due to a variety of measurement tools. Diverse methods were used for the TL analysis in our extracted studies, and the specimen types employed were also different. Generally, TL could differ depending on the tissue [42] and the analysis approach [43]. In this review, three out of five studies used the qPCR method for the evaluation of TL. qPCR has advantages because (1) TL can be measured even in small amounts of DNA and (2) the process is less labor-intensive. However, it has a limitation, since (1) there are variations between and within “batches” and (2) there is a lack of reference standards. The TRF method is the golden standard, but it has the cons of intensive labor and requires a large amount of DNA [44]. In addition, the whole blood and PBMCs have good DNA yield and quality, while salivary samples could destabilize DNA yields depending on temperature [45]. In the cross-sectional studies, the heterogeneity of the research results arose from the difference in the physical activity measurement tools that were used. The two cross-sectional studies analyzed the amount of physical activity using the Past Year Total Physical Activity Questionnaire (PYTPAQ) and the IPAQ short form, respectively. Since the amount of physical activity was measured using the questionnaires, the results may have been subject to self-reporting bias. Indeed, the contents of the questionnaires such as “physical activity domain” or “intensity”, and the recall period such as “during the past year” or “during the last 7 days”, could affect the amount of physical activity declared. Lee et al. [43] showed that questionnaires tend to overestimate the amount of physical activity performed by subjects compared to when an accelerometer is used. Therefore, more studies need to analyze the association between the intensity of physical activity and TL with an accelerometer.

The current study has several limitations. First, there is heterogeneity among the extracted studies. There are differences in the specimen, characteristics of participants, and variability of measurement tools in each study. Secondly, we should be cautious of generalizing the results, given that the included studies were exclusively published in English. Third, the method used to search the literature and retrieve from published articles may have caused reporting bias.

Despite the limitations, it is meaningful in that it confirmed the tendency of the association between physical activity or exercise and the TL of breast cancer survivors. Future studies need to clarify the effects of various confounding variables such as extracted tissues, characteristics of the participants, treatment modalities for breast cancer, and physical activity measurement tools. In addition, rigorous efforts are required to address existing challenges associated with TL sample storage and processing in all tissue types to ensure reproducibility and reliability of telomere samples and analytical methods.

## Figures and Tables

**Table 1:** Characteristics and quality assessment of included studies.

Author (Year)	Country	Study Design	Number of Subject	Specimen Type	Method of Evaluation of Telomere	Quality Assessment
Santa-Maria, C. A. et al. (2020) [21]	USA	Randomized controlled trial (RCT)	96 breast cancer survivors	Lymphocytes and granulocytes	Fluorescence in situ hybridization (FISH) assay	5
Sanft, T. et al. (2018) [24]	USA	Randomized controlled trial (RCT)	151 breast cancer survivors	Peripheral blood samples	Quantitative-polymerase chain reaction (qPCR), T/S	6
Ennour-Idrissi, K., et al. (2016) [27]	Canada	Cross-sectional study	164 women who underwent surgery for unilateral breast cancer	Peripheral white blood cells	Quantitative-polymerase chain reaction (qPCR), T/S	7
Garland, S. N. et al. (2014) [22]	USA	Cross-sectional study	392 postmenopausal women with stage I–III breast cancer	Peripheral blood mononuclear cells (PBMCs)	Terminal restriction fragment (TRF)	7
Qu, S. et al. (2013) [25]	China	Case-control study	1296 (601 incident breast cancer cases, 605 control)	Peripheral blood samples	Monochrome multiplex quantitative polymerase chain reaction (qPCR), T/S	6

PBMCs—peripheral blood mononuclear cell, qPCR—real-time quantitative PCR detecting system, TRF—terminal restriction fragment, T/S ratio—telomere (T), single copy gene (S) ratio T/S. Quality assessments were conducted using Newcastle-Ottawa Scale (NOS).

**Table 2:** The summary of the included randomized controlled trials studies.

Author (Year)	Subject	Age	Purpose of Intervention	Contents of Intervention	Duration	Result
Santa-Maria, C. A. et al. (2020) [21]	96 obese ^a^ breast cancer survivors with stage I–III breast cancer	Median (range) intervention 53 (33–71) self-directed 55 (30–73)	Weight loss	POWER-Remote - Frequency: weekly for 3 months, monthly for additional 9 months - Intensity: unknown - Type: telephone and web-based platform - Time: unknown	12-month	- **NS.** Change TL: 0.1 ± 0.7 in POWER-remote group, 0.1 ± 0.7 in self-directed group (*p* = 0.76)
Sanft, T. et al. (2018) [24]	151 obese ^a^ breast cancer survivors	Mean ± SD 57.8 ± 7.7	Weight loss	Weight loss intervention group (WL) ㆍDiet: reducing calorie intake ㆍPhysical activity - Frequency: individualized counseling sessions weekly - Intensity: moderate intensity - Type: combined - Time: 30 min	6-month	- **TL shortening in total: NS.** Change TL: −3% in WL vs. −5% in control (*p* = 0.12). - **TL shortening in stage 0/1: WL < Control** Change TL: −7% in WL vs. −8% in control (*p* = 0.01).

^a^ Body mass index ≥ 25 kg/m^2^, abbreviations: NS—non-significant, TL—telomere length.

**Table 3:** The summary of the included non-randomized controlled trials studies.

Author (Year)	Study Design	Subject	Age	PA Measurement Tool	Result
Ennour-Idrissi, K., et al. (2016) [27]	Cross-sectional study	162 women who underwent surgery for unilateral breast cancer	Mean ± SD 52.6 ± 7.9	- Past Year Total Physical Activity Questionnaire	- **PA↑ = TL↑** TPA (rs = 0.17, *p* = 0.033), occupational PA (rs = 0.15, *p* = 0.054) and transportation-related PA (rs = 0.19, *p* = 0.019).
Garland, S. N. et al. (2014) [22]	Cross-sectional study	392 postmenopausal women with stage I–III breast cancer	Mean ± SD 61.97 ± 10.36	- Physical activity: International Physical Activity Questionnaire (IPAQ)	- **TL shortening: No PA > MVPA** (mean 5.84 kb versus 6.11 kb; *p* = 0.006). - **PA↓ = TL↓** (Adjusted coefficient (Adj β) = −0.22; 95% CI, −0.41 to −0.03; *p* = 0.03)
Qu, S. et al. (2013) [25]	Case-control study	601 incident breast cancer cases 695 matched as controls	Mean ± SD 52.7 ± 8.8 in case 53.4 ± 9.0 in control	- Unknown	- **NS.** (exercise and telomere length *p* for interaction = 0.612)

Abbreviations: SD—standard deviation, NS—non-significant, TL—telomere length, PA—physical activity, TPA—total physical activity, MVPA—moderate to vigorous physical activity, CI—confidence interval, PA↑—high levels of physical activity, PA↓—low level of physical acitivity, TL↑—long TL, TL↓—short TL.

## Data Availability

Not applicable.

## Supplementary Materials

The following supporting information can be downloaded at: https://www.mdpi.com/article/10.3390/jcm11092527/s1, Table S1: Search strategy.
