# Supplementary material for: Association between Physical Activity and Telomere Length in Women with Breast Cancer: A Systematic Review
Source: J Clin Med. 2022 Apr 30;11(9):2527. doi: 10.3390/jcm11092527 (PMC9099544; doi:10.3390/jcm11092527)
Supplement: Supplementary file 1 [file jcm-11-02527-s001.zip › jcm-1642430-supplementary.pdf]

Table S1. Search strategy.

Ovid MEDLINE® 1946~		
#	**Searches**	**Results**
#1	exp Breast Neoplasms/	302900
#2	((cancer* or carcinoma* or neoplasm* or adenoma* or adenocarcinom* or tumour* or tumor* or malignan*) adj3 breast).mp.	413319
#3	1 or 2	413334
#4	exp Telomere/	15789
#5	Telomere*.mp.	27166
#6	4 or 5	27166
#7	3 and 6	578
#8	exp Exercise/	207054
#9	exp Exercise Therapy/	53868
#10	exp Sports/	190472
#11	exp Yoga/	3001
#12	(Exercise* or sport* or resistance or strengthen* or recreation or rehabilitat* or aerobics or running or jogging or swimming or walk* or Cycling or bycle* or Flexibility or Stretching or physical activit* or Yoga).mp.	2119896
#13	8 or 9 or 10 or 11 or 12	2156170
#14	7 and 13	62
#15	exp animals/ not humans.sh.	
#16	14 not 15	60
**EMBASE search strategy**		
#	**Searches**	**Results**
#1	'breast tumor'/exp	586710
#2	(breast NEAR/3 (cancer* OR carcinom* OR tumor* OR tumour* OR neoplasm* OR adeno* OR neoplasia* OR malignan*)):ti,ab	496227
#3	#1 OR #2	651169
#4	telomere'/exp	23536
#5	telomere*:ti,ab	28113
#6	#4 OR #5	34772
#7	#3 AND #6	1006
#8	'exercise'/exp OR 'kinesiotherapy'/exp OR 'sport'/exp OR 'yoga'/exp	549127

#9	(exercise*:ti,ab OR sport*:ti,ab OR resistance:ti,ab OR strengthen*:ti,ab OR recreation:ti,ab OR rehabilitat*:ti,ab OR aerobics:ti,ab OR running:ti,ab OR jogging:ti,ab OR swimming:ti,ab OR walk*:ti,ab OR cycling:ti,ab OR bycle*:ti,ab OR flexibility:ti,ab OR stretching:ti,ab OR physical:ti,ab) AND activit*:ti,ab OR yoga:ti,ab	585555
#10	#8 OR #9	1015597
#11	#7 AND #10	65
#12	'animals'/exp NOT ('humans'/exp AND 'animals'/exp)	5601706
#13	#11 NOT #12	64
**Cochrane Strategy**		
#	**Searches**	**Results**
#1	MeSH descriptor: [Breast Neoplasms] explode all trees	13386
#2	((cancer* or carcinoma* or neoplasm* or adenoma* or adenocarcinom* or tumour* or tumor* or malignan*) near/3 breast):ti,ab,kw (Word variations have been searched)	38244
#3	#1 or #2	38245
#4	MeSH descriptor: [Telomere] explode all trees	54
#5	(telomere*):ti,ab,kw (Word variations have been searched)	396
#6	#4 or #5	396
#7	#3 and #6	38
#8	MeSH descriptor: [Exercise] explode all trees	25218
#9	MeSH descriptor: [Exercise Therapy] explode all trees	14231
#10	MeSH descriptor: [Sports] explode all trees	15873
#11	MeSH descriptor: [Yoga] explode all trees	683
#12	((Exercise* or sport* or resistance or strengthen* or recreation or rehabilitat* or aerobics or running or jogging or swimming or walk* or Cycling or bycle* or Flexibility or Stretching or "physical activit*" or Yoga)):ti,ab,kw (Word variations have been searched)	300051
#13	#8 or #9 or #10 or #11 or #12	301105
#14	#7 and #13	17
**Web of Science search strategy**		
#	**Searches**	**Results**
#1	TS=(breast NEAR/3 (cancer* or carcinom* or tumor* or tumour* or neoplasm* or adeno* or neoplasia* or malignan*))	556848
#2	TS=(Telomere*)	29173

#3	TS=(Exercise* or sport* or resistance or strengthen* or recreation or rehabilitat* or aerobics or running or jogging or swimming or walk* or Cycling or bycle* or Flexibility or Stretching or "physical activit*" or Yoga)	556848
#4	#1 AND #2 AND #3	210
**ClinicalTrials.gov**		
**#**	**Find studies**	**Results**
	Recruitment status: All studies	
**Condition or disease**	Breast Cancer	
**Other terms**	telomere AND exercise	
	1 AND 2	4
